# Editorial: Glanders and melioidosis: one health model

**DOI:** 10.3389/fvets.2023.1303556

**Published:** 2023-10-13

**Authors:** Harisankar Singha, Apichai Tuanyok, Mandy Elschner, Karine Laroucau, Chiranjay Mukhopadhyay

**Affiliations:** ^1^ICAR-National Research Centre on Equines, Hisar, India; ^2^Department of Infectious Diseases and Immunology, College of Veterinary Medicine, University of Florida, Gainesville, FL, United States; ^3^Institute of Bacterial Infections and Zoonoses, Friedrich Loeffler Institute, Jena, Germany; ^4^French Agency for Food, Environmental and Occupational Health & Safety (ANSES), Maisons-Alfort, France; ^5^Kasturba Medical College, Manipal Academy of Higher Education, Manipal, India; ^6^Center for Emerging and Tropical Diseases, Manipal Academy of Higher Education, Manipal, India

**Keywords:** One Health, glanders, melioidosis, zoonosis, vaccine, antibiotics, genotyping

Glanders and melioidosis are both neglected and emerging diseases, endemic in regions with low and middle-income economies. These diseases are caused by closely related Gram-negative bacteria named *Burkholderia (B.) mallei* and *B. pseudomallei*, respectively. These bacteria are classified as Tier 1 select agents by the US Federal Select Agent Program because of their fatality, biothreat potential, lack of vaccines, and intrinsic antibiotic resistance characteristics.

In nature, *B. pseudomallei* can survive both as a free-living organism within environmental niches, such as soil and water, and as a parasite living in host organisms, such as amoeba, plants, and fungi (1). This ubiquity within the environment heightens the potential for infection across a spectrum of species, including humans and animals, with livestock like goats, pigs, and sheep being commonly affected, along with wildlife. On the other hand, *B. mallei* requires a natural host, primarily equines, for its survival and subsequent transmission to susceptible animals, including humans. The transmission routes (ingestion, inhalation, or cutaneous abrasion) and clinical manifestations exhibit striking similarities between the two diseases. These life-threatening diseases present a wide range of non-specific signs and symptoms, including fever, pneumonia, acute septicemia, and chronic localized infection. Chronic infection can cause abscesses in various internal organs, such as the lungs, liver, spleen, kidneys, prostate gland, and skeletal muscles.

Glanders has been eradicated from the majority of the developed countries, including Europe, Northern Americas, Australia, and New Zealand; however, the disease is still prevalent in South East Asia, the Middle-East, Latin America and parts of Africa (2). In contrast, melioidosis is prevalent in different countries, such as Thailand, Australia, Vietnam, Laos, Cambodia, Myanmar, India, Sri Lanka, and Bangladesh (3). Both glanders and melioidosis exemplify the “One Health” concept due to their impact on the well-being of humans, domestic animals, wildlife, and ecosystems. These diseases are recognized as major threats within endemic areas and are capable of spreading to non-endemic areas. Hence, there is an imperative need to know more about the epidemiology, pathogenesis, treatment and prophylaxis aspects of these two closely related organisms that cause serious life-threatening diseases and have biothreat potential. “One Health” is a collaborative approach that aims to improve health for people, animals, plants, and the environment. However, in region with limited resources, the lack of proper systems to monitor and report cases of glanders and melioidosis can make difficult to quickly detect and control outbreak events.

There are seven articles for this Research Topic, including five original studies and two review articles which focus on relevant areas of the diseases like treatments, vaccines, surveillance, and epidemiology (Table 1). Barnes, Bayliss et al. and Barnes, Richards et al. submitted two research articles on treatment of melioidosis and glanders. Doxycycline was down selected from a panel of antibiotics evaluated *in vitro* and used in combination with finafloxacin in a mouse model of inhalational melioidosis. A combination of doxycycline and finafloxacin oral therapy improve survival and the clearance of colonizing bacteria from tissues and, in addition, reduce the potential of relapse of infection. In the second paper, the fluoroquinolone finafloxacin was compared to co-trimoxazole as a post-exposure prophylactic in a murine model of inhalational glanders. Although no difference in protection was observed between finafloxacin and co-trimoxazole, finafloxacin treatment appeared to provide better clearance of bacteria in organs and control the development of clinical signs of disease. Klimko et al., combined vaccination strategies (protein subunit or live attenuated) and co-trimoxazole regimens against melioidosis. It was demonstrated that partially protective *B. pseudomallei* vaccination strategies can synergize with suboptimal antibiotic regimens resulting in nearly 100% survival in a mouse model of melioidosis. Biryukov et al., evaluated the humoral and cell-mediated immune response and protective efficacy of *Burkholderia* vaccine candidates against lethal aerosol challenges with *B. pseudomallei* and *B. mallei* in a mouse model. A protein subunit vaccine and an attenuated *B. pseudomallei* strain, provided significant protection.

**Table 1:** Summary of the published articles in the Research Topic related to “*Glanders and melioidosis: one health model*.”

**Sr No**.	**Article title**	**Key findings**
1	Efficacy of finafloxacin in a murine model of inhalational glanders (Barnes, Bayliss et al.)	In this study, the fluoroquinolone finafloxacin was compared to co- trimoxazole as a post-exposure prophylactic in a murine model of inhalational glanders. Although no difference in protection was observed between finafloxacin and co-trimoxazole, finafloxacin treatment appeared to provide better clearance of bacteria in organs and control the development of clinical signs of disease.
2	Investigation of a combination therapy approach for the treatment of melioidosis (Barnes, Richards et al.)	This manuscript focuses on an antibiotic combination approach to treat melioidosis. Doxycycline was down selected from a panel of antibiotics evaluated *in vitro* and used in combination with finafloxacin in a Balb/c mouse model of inhalational melioidosis. A combination of doxycycline and finafloxacin oral therapy improve survival and the clearance of colonizing bacteria from tissues and, in addition, reduce the potential of relapse of infection.
3	Layered and integrated medical counter- measures against *Burkholderia pseudomallei* infections in C57BL/6 mice (Klimko et al.)	In this manuscript combined vaccination strategies (protein subunit or live attenuated) and co-trimoxazole regimens were evaluated against melioidosis. It was demonstrated that partially protective *B*. *pseudomallei* vaccination strategies can synergize with suboptimal antibiotic regimens resulting in nearly 100% survival in a C57BL/6 mouse model of melioidosis.
4	Evaluation of two different vaccine platforms for immunization against melioidosis and glanders (Biryukov et al.)	The study evaluated the humoral and cell-mediated immune response and protective efficacy of three Burkholderia vaccine candidates against lethal aerosol challenges with *B. pseudomallei* K96243, *B. pseudomallei* MSHR5855, and *B. mallei* FMH in the C57BL/6 mouse model. Two vaccines, a capsule conjugate+Hcp1 subunit vaccine and the live *B. pseudomallei* 668 ΔilvI strain, provided significant protection.
5	One Health surveillance approaches for melioidosis and glanders: the Malaysian perspective (Mariappan et al.)	This review aims to define the organizational setup and functional characteristics of One Health surveillance approaches for glanders and melioidosis. It was suggested that well-planned control programs and campaigns along with commitment, strong political support, and adequate resources are key to the success of surveillance and controlling both diseases in Malaysia.
6	*Burkholderia pseudomallei* in soil and natural water bodies in rural Sri Lanka: a hidden threat to public health (Jayasinghearachchi et al.)	This study reports the isolation of eight *B. pseudomallei* from soil and natural water in the western, northwestern and southern parts of Sri Lanka around the homes and workplaces of melioidosis patients. The Sri Lankan environmental isolates clustered with the Australian isolates.
7	Sequence-based detection and typing procedures for Burkholderia mallei: Assessment and prospects (Brangsch et al.)	This review illustrated the need of refinement of the presently used molecular typing methods in perspective of high homology and clonality of *B. mallei* genome. Availability of large number of accurate *B. mallei* genome sequence and single nucleotide polymorphism (SNP) based techniques like HRM-PCR and core genome multi locus sequence typing (cgMLST) would contribute to the greater typing resolution and understanding of *B. mallei* global genotype distribution.

Jayasinghearachchi et al. conducted an environmental surveillance, focusing on soil and natural water sources suspected to harbor *B. pseudomallei*. Their study reports the isolation of eight *B. pseudomallei* strains, situated in proximity to the residences and workplaces of melioidosis patients. These environmental isolates clustered with Australian isolates. Consequently, to mitigate the risk of melioidosis transmission, it is recommended to implement preventive measures such as well chlorination, water filtration, or boiling prior to consumption.

This Research Topic also includes two comprehensive reviews. The initial review, by Mariappan et al., discussed “One Health” surveillance approaches for melioidosis and glanders, contextualized within the Malaysian perspective. This review aims to define the organizational setup and functional characteristics of “One Health” surveillance approaches for glanders and melioidosis. It was suggested that well-planned control programs and campaigns, along with commitment, strong political support, and adequate resources, are key to the success of surveillance and control measures for both diseases in Malaysia. The second review, by Brangsch et al., focused on different molecular techniques based on allelic discrimination, such as MLST, VNTR, restriction enzyme digestion (PFGE), single nucleotide polymorphism (HRM-PCR and cgMLST), and highlighted their advantages and disadvantages for accurately typing of *B. mallei* strains isolated in different countries. The authors have opined that advent and continuous advancement of the sequencing techniques as well as reconstruction of incorrect *B. mallei* genomes in public repositories would be helpful to enable the development of genome-guided epidemiological tools.

In summary, glanders and melioidosis pose a significant threat to animal and public health, necessitate the adoption of a concerted “One Health” strategy. Enhancements in diagnostic techniques, enabling more accurate detection, along with progress in drug discovery and vaccine development, are of importance in addressing the diagnosis and treatment of infections in both humans and animals. We expect that this compilation will provide new exciting insights into medical countermeasures, “One Health” surveillance, and molecular epidemiology on those two disease, which are emerging and fatal, but still “neglected.”

## Author contributions

CM: Conceptualization, Writing—original draft, Writing—review and editing. HS: Conceptualization, Formal analysis, Writing—original draft, Writing—review and editing. AT: Conceptualization, Supervision, Writing—review and editing. ME: Conceptualization, Supervision, Writing—review and editing. KL: Conceptualization, Supervision, Writing—review and editing.

## References

[1] Duangurai T Indrawattana N Pumirat P. Burkholderia pseudomallei Adaptation for Survival in Stressful Conditions. Biomed Res Int. (2018) 2018:3039106. 10.1155/2018/3039106 29992136 PMC5994319

[2] Singha H Shanmugasundaram K Tripathi BN Saini S Khurana SK Kanani A. Serological surveillance and clinical investigation of glanders among indigenous equines in India from 2015 to 2018. Transbound Emerg Dis. (2020) 67:1336–1348. 10.1111/tbed.13475 31916415

[3] Mukhopadhyay C Shaw T Varghese GM Dance DAB. Melioidosis in South Asia (India, Nepal, Pakistan, Bhutan and Afghanistan). Trop Med Infect Dis. (2018) 3:51. 10.3390/tropicalmed3020051 30274447 PMC6073985

